# The *TP53* gene rs1042522 C>G polymorphism and neuroblastoma risk in Chinese children

**DOI:** 10.18632/aging.101196

**Published:** 2017-03-08

**Authors:** Jing He, Fenghua Wang, Jinhong Zhu, Zhuorong Zhang, Yan Zou, Ruizhong Zhang, Tianyou Yang, Huimin Xia

**Affiliations:** ^1^ Department of Pediatric Surgery, Guangzhou Institute of Pediatrics, Guangzhou Women and Children's Medical Center, Guangzhou Medical University, Guangzhou 510623, Guangdong, China; ^2^ Sun Yat-sen University Cancer Center, State Key Laboratory of Oncology in South China, Department of Experimental Research, Collaborative Innovation Center for Cancer Medicine, Guangzhou 510060, Guangdong, China; ^3^ Molecular Epidemiology Laboratory and Department of Laboratory Medicine, Harbin Medical University Cancer Hospital, Harbin 150040, Heilongjiang, China

**Keywords:** *TP53*, neuroblastoma, rs1042522, polymorphism, susceptibility

## Abstract

*TP53*, a tumor suppressor gene, plays a critical role in cell cycle control, apoptosis, and DNA damage repair. Previous studies have indicated that the *TP53* gene Arg72Pro (rs1042522 C>G) polymorphism is associated with susceptibility to various types of cancer. We evaluated the association of the *TP53* gene rs1042522 C>G polymorphism with neuroblastoma susceptibility in a hospital-based study among the Chinese Han population. Enrolled were 256 patients and 531 controls. Odds ratios (ORs) and 95% confidence intervals (CIs) generated using logistic regression models were used to determine the strength of the association of interest. No association was detected between rs1042522 C>G polymorphism and neuroblastoma risk. In our stratification analysis of age, gender, sites of origin, and clinical stages, we observed that subjects with rs1042522 CG/GG genotypes had a lower risk of developing neuroblastoma in the mediastinum (Adjusted OR=0.52, 95% CI=0.33-0.82, *P*=0.005) than those carrying the CC genotype. These results indicate that *TP53* gene rs1042522 C>G polymorphism may exert a weak and site-specific effect on neuroblastoma risk in Southern Chinese children and warrant further confirmation.

## INTRODUCTION

Neuroblastoma, a common pediatric solid tumor, accounts for about 10% of all childhood cancers, and is the third leading cause of cancer-related death in children [1]. The incidence rate of neuroblastoma is relatively low, approximately affecting 7.7 live births per million in China [2]. However, incidence is higher in Europe and the United States, with 8-14 cases per million [3]. Neuroblastoma is a cancer of the sympathetic nervous system with diverse clinical phenotypes [4]. A substantial proportion of neuro-blastomas are localized benign tumors that can spontaneously regress and thus patients bearing these tumors may have a favorable prognosis [5]. However, approximately 50% of patients show an aggressive clinical course with a survival rate of less than 35% despite aggressive therapy [4, 6].

The exact etiology and pathogenesis of neuroblastoma remains unknown [7]. It has been reported that offspring can be more susceptible to neuroblastoma if the parents were exposed to risk factors such as wood dust, radiation sources, and hydrocarbons [8, 9]. On the contrary, parental exposure to risk factors alone cannot explain the phenomenon that only a small portion of affected offspring develops neuroblastoma. Growing evidence indicates that genetic polymorphisms increase predisposition to neuroblastoma [10, 11]. In addition to genome-wide association studies (GWASs), candidate gene approaches have also been applied to identify potential variants associated with neuroblastoma. Indeed, several neuroblastoma predisposition genes have been discovered, including *FAS* [12], *FASL* [12], *NEFL* [11], *TGFBR3L* [13], *XPG* [14] and *XPC* [15].

Tumor suppressor gene *TP53* is located on chromosome 17p13. p53, a protein encoded by the *TP53* gene, plays a pivotal role in cell cycle control, apoptosis, senescence, and maintenance of DNA integrity [16-18] by regulating the expression of many genes including *p21*, *PUMA*, *DRAM*, and *MDM2*, among others [19]. The *TP53* gene is one of the most frequently mutated genes in human cancers [16, 20-23]. More than 200 single nucleotide polymorphisms (SNPs) have been reported in the *TP53* gene [22]. A non-synonymous polymorphism leading to the substitution of proline for arginine (Arg72Pro) at codon 72 of the p53 protein was discovered in the *TP53* gene (rs1042522 G>C) [24]. Multiple studies have been performed among populations of different ethnic background to investigate the association between this functional *TP53* polymorphism and the risk of many cancers, including cervical cancer, colorectal cancer, breast cancer, lung cancer, ovarian cancer, and endometrial cancer [25-29]. However, few studies have focused on neuroblastoma. Here, we performed a hospital-based case-control study using data from 256 neuroblastoma patients and 531 control subjects to evaluate the association between the *TP53* gene rs1042522 G>C polymorphism and neuro-blastoma risk in Southern Chinese children.

## RESULTS

### Population characteristics

Our research population consisted of 256 neuroblastoma patients and 531 cancer-free controls. The demographic characteristics of all participants are shown in Supplemental Table 1. There was no significant difference in age (*P*=0.239) nor gender (*P*=0.333) between cases and controls. Regarding the sites of tumor origin, 46 (17.97%), 87 (33.98%), 90 (35.16%), 25 (9.77%) neuroblastomas occurred in adrenal glands, retroperitoneal region, mediastinum, and other regions, respectively, while the origin of eight (3.13%) neuroblastomas was not determined because of the limited availability of tumor samples. Moreover, according to the INSS criteria [30], 54 (21.09%), 65 (25.39%), 44 (17.19%), 77 (30.08%), and nine (3.52%) cases were classified into stage I, II, III, V, and 4s disease, respectively, with an exception of seven cases (2.73%) classified into NA (not available) due to lack of information.

### *TP53* gene rs1042522 C>G polymorphism with neuroblastoma risk

The frequency of occurrence of the *TP53* gene rs1042522 C>G genotype in cases and controls, as well as the associations with neuroblastoma risk, are listed in Table 1. Our observations agree with Hardy-Weinberg equilibrium conditions (*P*=0.440) among the controls. The genotype frequency distribution of the *TP53* gene rs1042522 C>G polymorphism was as follows: 35.55% (CC), 42.19% (CG) and 22.27% (GG) in the patients and 29.25% (CC), 48.11% (CG) and 22.64% (GG) in the controls. No association between the rs1042522 C>G polymorphism and neuroblastoma risk was observed, even when age and gender were adjusted for.

**Table 1:** Genotype distributions of *TP53* gene rs1042522 C>G polymorphism and neuroblastoma susceptibility

Genotype	Cases (N=256)	Controls (N=530)	*P* ^a^	Crude OR (95% CI)	*P*	Adjusted OR (95% CI) ^b^	*P* ^b^
rs1042522 (HWE=0.440)							
CC	91 (35.55)	155 (29.25)		1.00		1.00	
CG	108 (42.19)	255 (48.11)		0.72 (0.51-1.02)	0.062	0.72 (0.51-1.02)	0.065
GG	57 (22.27)	120 (22.64)		0.81 (0.54-1.22)	0.309	0.80 (0.53-1.21)	0.290
Additive			0.175	0.88 (0.72-1.08)	0.229	0.88 (0.72-1.08)	0.215
Dominant	165 (64.45)	375 (70.75)	0.076	0.75 (0.55-1.03)	0.075	0.75 (0.55-1.03)	0.074
Recessive	199 (77.73)	410 (77.36)	0.906	0.98 (0.68-1.40)	0.906	0.97 (0.68-1.39)	0.860

OR, odds ratio; CI, confidence interval, HWE, Hardy-Weinberg equilibrium.

a *χ^2^* test for genotype distributions between neuroblastoma patients and controls.

b Adjusted for age and gender.

### Stratification analysis

We further explored the association between rs1042522 C>G polymorphism and neuroblastoma susceptibility stratifying by age, gender, tumor sites, and clinical stages. As shown in Table 2, when compared with the CC genotype, the CG/GG genotypes of the rs1042522 C>G polymorphism were associated with a decreased risk of developing neuroblastoma in mediastinum [adjusted odds ratio (OR) =0.52, 95% confidence interval (CI)=0.33-0.82, *P*=0.005]. Moreover, we also observed a borderline significant protective association between the rs1042522 C>G polymorphism and neuroblastoma risk in males (adjusted OR=0.66, 95% CI=0.44-1.00, *P*=0.051). No other significant associations were detected.

**Table 2:** Stratification analysis for the association between *TP53* gene rs1042522 C>G polymorphism and neuroblastoma susceptibility

Variables	rs1042522 (cases/controls)		Crude OR	*P*	Adjusted OR ^a^	*P* ^a^
	CC	CG/GG	(95% CI)		(95% CI)	
Age, month						
≤18	36/68	65/165	0.74 (0.45-1.22)	0.242	0.74 (0.45-1.21)	0.229
>18	55/87	100/210	0.75 (0.50-1.14)	0.179	0.76 (0.50-1.14)	0.186
Gender						
Females	34/71	69/161	0.90 (0.55-1.47)	0.661	0.89 (0.54-1.47)	0.649
Males	57/84	96/214	0.66 (0.44-1.00)	0.0499	0.66 (0.44-1.00)	0.051
Sites of origin						
Adrenal glands	13/155	33/375	1.05 (0.54-2.05)	0.888	1.04 (0.53-2.04)	0.906
Retroperitoneal	29/155	58/375	0.83 (0.51-1.34)	0.440	0.82 (0.51-1.34)	0.430
Mediastinum	40/155	50/375	**0.52 (0.33-0.82)**	**0.005**	**0.52 (0.33-0.82)**	**0.005**
Other	7/155	18/375	1.06 (0.44-2.60)	0.894	1.06 (0.43-2.59)	0.899
Clinical stages						
I+II+4s	44/155	75/375	0.70 (0.46-1.07)	0.099	0.71 (0.47-1.07)	0.104
III+IV	40/155	81/375	0.84 (0.55-1.28)	0.409	0.84 (0.55-1.28)	0.417

OR, odds ratio; CI, confidence interval.

a Adjusted for age and gender, omitting the corresponding stratification factor.

## DISCUSSION

In our hospital-based case-control study here, we explored the relationship between the *TP53* gene rs1042522 C>G polymorphism and neuroblastoma susceptibility. However, we did not detect a main effect on neuroblastoma susceptibility for the rs1042522 C>G polymorphism. Overwhelming evidence suggests that the *TP53* gene is a crucial tumor suppressor. Disruption or abnormally low transcription of the *TP53* gene can impair the tumor-suppressing function of the p53 signaling pathway, thereby promoting tumor development and progression [20]. The *TP53* gene is the most frequently mutated gene in many human cancers [31, 32]. The *TP53* gene rs1042522 C>G polymorphism in exon 4 results in a non-conservative transversion of arginine (Arg) to proline (Pro) at codon 72 [33]. It is the most commonly studied genetic variant in the *TP53* gene, and its implications in cancer genetic epidemiology have been amply documented [34-36]. Previous functional analyses indicated that the two alleles of the *TP53* gene have differential capacities to regulate various cellular functions. For example, the p53 codon 72 Pro variant exhibits a markedly reduced capacity to induce apoptosis compared to wild-type p53 due to decreased mitochondria localization [37], but an increased efficiency in the induction of cell cycle arrest [38].

Several studies have indicated that the *TP53* gene rs1042522 C>G polymorphism might promote tumor development; however, consensus has not been reached. Khan et al. genotyped 140 thyroid cancer patients and 200 cancer-free controls from Kashmir Valley to evaluate the association between the *TP53* gene rs1042522 C>G polymorphism and the risk of differentiated thyroid cancer [38]. They found that rs1042522 C>G polymorphism conferred higher susceptibility to thyroid cancer [39]. In a study conducted among the Bangladeshi population including 50 histopatho-logically confirmed lung cancer patients and 50 age-matched controls, Chowdhury *et al.* found that the *TP53* gene rs1042522 CC genotype is a risk factor for lung cancer [40]. Wu *et al*. also suggested that there is an increased risk of gastric cancer for individuals with the *TP53* gene rs1042522 C>G polymorphism among the Chinese Han population [41]. However, in another case-control study analyzing the association between the *TP53* gene rs1042522 C>G polymorphism and retinoblastoma risk in the Chinese Han population, Chen *et al*. found that no in allele or genotypic frequencies of the *TP53* gene rs1042522 C>G between cases (n=168) and controls (n=185) [42].

Although many studies have been carried out to investigate the association between the *TP53* gene rs1042522 C>G polymorphism and the risk of various cancers, only two of them addressed neuroblastoma. Cattelani *et al*. conducted the first of these studies in a population of European descent. They found that the *TP53* gene rs1042522 C>G polymorphism had no impact on the risk of developing neuroblastoma in 288 healthy subjects and 286 neuroblastoma patients [43]. By analyzing three independent case-control cohorts comprising 10,290 individuals, Diskin et al. found that the *TP53* gene rs78378222 A>C and rs35850753 A>G polymorphisms were robustly associated with neuro-blastoma risk. However, they failed to detect any association between the *TP53* gene rs1042522 C>G variant and overall survival in 1,809 neuroblastoma patients [44]. In addition, very few studies have found a relationship between other polymorphisms in the *TP53* gene and neuroblastoma risk. E.g., Rihani *et al.* investigated the impact of rs1042522 C>G and rs78378222 A>C in the *TP53* gene, but failed to detect any significant relationship with neuroblastoma risk [45].

Our current study shows that the rs1042522 C>G polymorphism might not affect the susceptibility to neuroblastoma in most patients. However, more studies are needed to further substantiate our negative observation due to multiple factors limiting our study. For example, our study could not have detected the possible mild effects of low-penetrating genetic variants because of our small sample size due to the low incidence of this disease. Indeed, the modest association we measured between the rs1042522 C>G poly-morphism and neuroblastoma risk in the mediastinum subgroup may be attributable to the relatively small sample size of this study. Furthermore, some susceptibility alleles in single genes may moderately contribute to neuroblastoma risk. In addition, neuroblastoma is a multi-factorial disease resulting from multiplicative interactions between environmental factors and genetic backgrounds. Our study lacked information on some valuable parameters, such as parental exposures, dietary intake, and living environment. Indeed, interacting factors, such as environmental exposures or interfering genes (*MDM2*, *MDM4* and *Hausp*), may override the effects of the rs1042522 C>G polymorphism [46]. Furthermore, selection bias might also exist, since our study was a hospital-based study with subjects recruited from southern China; therefore, our study population might not be representative of the genera Chinese population. Finally, only the rs1042522 C>G polymorphism was included in this study. However, it is clear that only a small proportion of SNPs can influence cancer susceptibility (driver mutation), while most of them cannot (passenger mutations) [47]. Therefore, discovering or discarding any potential influence of the rs1042522 C>G polymorphism on neuroblastoma susceptibility requires further studies with larger populations, including other functional SNPs. Nonetheless, our study is the largest analyzing the correlation between the *TP53* gene rs1042522 C>G polymorphism and neuroblastoma risk among the Chinese population. We found that the *TP53* gene rs1042522 C>G polymorphism had no main effect on neuroblastoma susceptibility. However, since the effect of this polymorphism might be influenced by site and sex, further prospective, large-scale, multicenter studies involving populations of different ethnicities are required to strengthen our findings.

## MATERIALS AND METHODS

### Study subjects

The subjects recruited were described in previous studies [14, 48-50]. Briefly, all of them were genetically unrelated ethnic Han Chinese from southern China. This study included 256 patients with newly diagnosed and histologically confirmed neuroblastoma and 531 cancer-free controls. The current study was approved by the Institutional Review Board of Guangzhou Women and Children's Medical Center. Informed written consent was obtained from participants’ parents or their legal guardians.

### Genotyping by Taqman

The *TP53* gene rs1042522 C>G was genotyped by using Taqman real-time PCR on a 7900 Sequence Detection System (Applied Biosystems, Foster City, CA), as previously described [51-53]. For quality control purposes, eight duplicate positive controls and eight negative controls without DNA were used in each of 384-well plates.

### Statistical analysis

Goodness-of-fit χ^2^ test was performed to test for deviations from Hardy-Weinberg equilibrium in genotype frequencies of the polymorphism in controls. Two-sided χ^2^ test was used to evaluate the differences in demographic variables and frequency distributions of genotype between patients and controls. We conducted unconditional univariate logistic regression to estimate the association between the *TP53* gene rs1042522 C>G polymorphism and neuroblastoma susceptibility by computing ORs and 95% 95% CIs. Adjusted ORs were calculated using multivariate analysis adjusting for age and gender. All statistical analyses were performed using SAS software (version 9.1; SAS Institute, Cary, NC). *P* < 0.05 was considered as statistically significant.

## SUPPLEMENTARY MATERIALS TABLE


