# Supplementary material for: The TP53 gene rs1042522 C>G polymorphism and neuroblastoma risk in Chinese children
Source: Aging (Albany NY). 2017 Mar 8;9(3):852–8. doi: 10.18632/aging.101196 (PMC5391235; doi:10.18632/aging.101196)
Supplement: Supplementary file 1 [file aging-09-852-s001.pdf]

SUPPLEMENTARY MATERIAL

Supplemental Table 1. Frequency distribution of selected variables for neuroblastoma cases and controls

Variables	Cases (n=256)		Controls (n=531)		*P* [a]
	No.	%	No.	%	
Age range, month	0-156		0.07-156		0.239
Mean ± SD	30.87 ± 26.45		29.73 ± 24.86		
≤18	101	39.45	233	43.88	
>18	155	60.55	298	56.12	
Gender					0.333
Female	103	40.23	233	43.88	
Male	153	59.77	298	56.12	
Clinical stages					
I	54	21.09			
II	65	25.39			
III	44	17.19			
IV	77	30.08			
4s	9	3.52			
NA	7	2.73			
Sites of origin					
Adrenal glands	46	17.97			
Retroperitoneal region	87	33.98			
Mediastinum	90	35.16			
Other regions	25	9.77			
NA	8	3.13			

SD, standard deviation; NA, not available.

[a] Two-sided χ^2 test for the distributions between neuroblastoma cases and controls.
